# Further Insights on Honey and Propolis from Gerês (Portugal) and Their Bioactivities: Unraveling the Impact of Beehive Relocation

**DOI:** 10.3390/life14040506

**Published:** 2024-04-15

**Authors:** Ana Sofia Freitas, Rui Oliveira, Cristina Almeida-Aguiar

**Affiliations:** 1 CITAB—Centre for the Research and Technology of Agro-Environmental and Biological Sciences, University of Minho, *Campus* de Gualtar, 4710-057 Braga, Portugal; anasofiapfreitas@gmail.com; 2 Department of Biology, School of Sciences, University of Minho, *Campus* de Gualtar, 4710-057 Braga, Portugal; 3 CBMA—Centre of Molecular and Environmental Biology, University of Minho, *Campus* de Gualtar, 4710-057 Braga, Portugal

**Keywords:** beehive relocation, propolis, honey, antibacterial activity, antioxidant potential

## Abstract

Propolis, a bee product, is known for its variability of chemical and bioactive profiles. However, Portuguese propolis from Gerês, normally obtained by mixing propolis from three places—Bugalho, Felgueiras and Toutelo—has shown similar chemical and biological profiles over the years. Recently, a new propolis place—Roca—was added to the apiary to replace Bugalho, lost to the 2017 wildfires, hence questioning the previously claimed constancy of Gerês propolis. To unravel to what extent the beehive relocation affected this constancy, we studied different Gerês propolis samples collected in three consecutive years (2017–2019) composed of different combinations of source places. Two honey samples, collected before (2017) and after (2018) the occurrence of the wildfire, were also investigated. Total phenolics, flavonoids and *ortho*-diphenols contents were determined and the antioxidant and antimicrobial activities were evaluated, using the DPPH assay and the agar dilution method, respectively. Although both antimicrobial and antioxidant activities were generally in the ranges usually obtained from Gerês propolis, some variations were detected for the samples, with different compositions when compared to previous years. This work reinforces the importance of the consistency of a combination of several factors for the protection and preservation of the flora near the hives, providing bee products with more constant chemical and biological profiles over the years.

## 1. Introduction

Propolis, or bee glue, is a complex mixture composed of resinous and balsamic materials collected by honeybees from branches, flowers, pollen, buds and exudates of trees, which are mixed with salivary enzymes and other compounds resulting from bees’ metabolism [1,2]. Propolis is already recognized as an important source of bioactive compounds, with properties for several applications [3,4,5]. The chemical and biological profiles of this beehive product are usually very variable according to several factors such as the source plant species [6,7], climate characteristics [7], harvesting time [8] and technique, bee species [9], solvent, and method of extraction [10,11]. However, propolis studied in this work is peculiar due to an unusual constancy of its chemical and biological profiles reported for the period between 2011 and 2016, which was linked to its production in an organic mode, standardized method of collection (from grids), and the consistency of the vegetation nearby the apiary [12], which is located in a protected area of the Peneda—Gerês National Park.

Plant diversity defines different types of propolis, containing numerous but also diverse chemical constituents responsible for its biological properties, which include antimicrobial, antioxidant and antitumor properties, among many others [4,13,14]. Such biological properties are thought to be connected to the complex chemical composition, involving a mechanism putatively attributed to synergistic effects between the chemical compounds [15]. Among the 850 compounds already identified in different propolis samples [16], phenolics seem to be the most important. Flavonoids (flavones, flavonols and flavonones), phenolic acids and their esters are the main bioactive compounds found in European propolis [17], with the flavonoids pinocembrin, galangin and chrysin and the phenolic acids caffeic acid, ferulic acid and cinnamic acid being the most common phenolic compounds in propolis from temperate zones [17,18].

Propolis from Gerês is usually composed of a mixture of propolis collected from beehives located at different places in Gerês: Bugalho, Felgueiras and Toutelo. However, in 2017, wildfires destroyed one of these source places—Bugalho—and, despite the installation of new beehives at the same location in 2018, allowing propolis samples similar to those of previous years to be obtained, the swarms did not resist, and the beehives had to be moved to another location—Roca. Subsequently, in order to search for the constancy usually observed in Gerês propolis [12], which has been questioned by the beehive relocation, we studied eleven ethanol extracts of propolis sampled from Gerês collected in three consecutive years (2017–2019) composed of different combinations of source places—Bugalho, Felgueiras, Toutelo and Roca. Two honey samples—collected before (2017) and after (2018) the occurrence of the wildfire—were included in the present study too. Pollen analysis, as well as the chemical characterization and the antimicrobial and antioxidant activities of propolis and honey, were investigated and compared with samples from Gerês collected in previous years.

## 2. Materials and Methods

### 2.1. Propolis and Honey Samples and Extract Preparation

**Figure 1:**
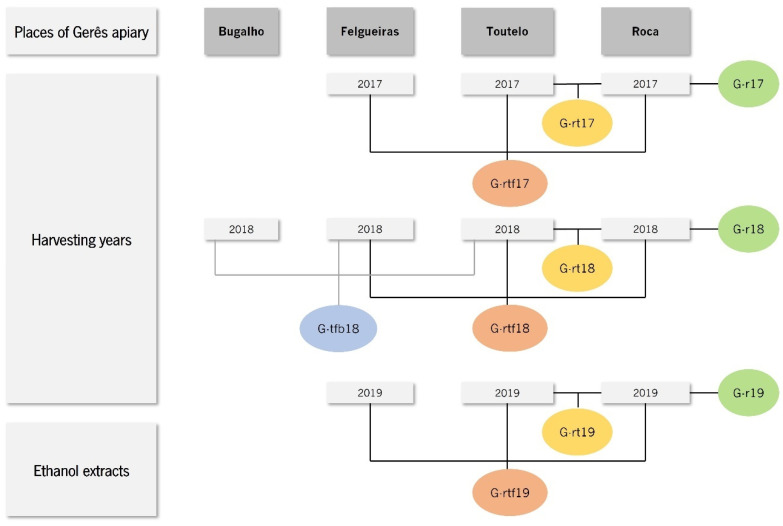
Propolis was collected from different places of Gerês apiary—Bugalho (G-b), Felgueiras (G-f), Toutelo (G-t) and Roca (G-r)—over three consecutive years. Propolis samples included different combinations of propolis harvested each year (2017, 2018 and 2019): Roca (G-r), Roca with Toutelo (G-rt), Toutelo with Felgueiras (G-tf), Toutelo with Felgueiras and Bugalho (G-tfb), and Roca with Toutelo and Felgueiras (G-rtf). Ethanol extraction of these samples resulted in the following eleven extracts: G-r17.EE, G-rt17.EE and G-tf17.EE (of 2017); G-r18.EE, G-rt18.EE, G-tf18.EE and G-tfb18.EE (same as G18.EE) (of 2018); and G-r19.EE, G-rt19.EE, G-tf19.EE and G-rtf19.EE (same as G19.EE) (of 2019).

The propolis and honey samples used in this work were harvested from an apiary located in Gerês (G) in the north of Portugal and near the Cávado River (41°45′41.62″ N; 7°58′03.34″ W). Propolis was collected from the different available places of Gerês apiary—Bugalho (G-b), Felgueiras (G-f), Toutelo (G-t) and Roca (G-r)—over three consecutive years (Figure 1). Ethanol extraction was performed as previously described [12] from samples resulting from different combinations of propolis collected each year (Figure 1). Briefly, a volume of 80 mL of ethanol was added to approximately 15 g of each propolis sample and the mixtures were placed under orbital agitation (100 rpm), at 24 °C, in the dark, for 24 h. Mixtures were then filtered under a vacuum and the filtrates were reserved at 4 °C. The solid residues were extracted again with 50 mL of the same solvent. The resulting filtrates were pooled and dried in a Buchi Rotavapor RE 121 (40 rpm, at 40 °C), yielding the dried ethanol extracts of propolis from Gerês (11 G.EEs): G-r17.EE, G-rt17.EE and G-tf17.EE of 2017; G-r18.EE, G-rt18.EE, G-tf18.EE and G-tfb18.EE (the same as G18.EE) of 2018; and G-r19.EE, G-rt19.EE, G-tf19.EE and G-rtf19.EE (the same as G19.EE) of 2019 (Figure 1). The eleven G.EEs were stored at 4 °C in the dark until experimental use. Stock solutions of propolis extracts were prepared by dissolving each G.EE in absolute ethanol to obtain the desired working concentrations. 

Honey samples (H), harvested in 2017 (H17) and 2018 (H18), were diluted in water (W) to a stock solution (S) of 50% (*v*/*v*)—H17.WS and H18.WS—to be used in the following described assays.

### 2.2. Botanical Origin Analysis

Pollen samples collected during two consecutive years (2017 and 2018) by the beekeeper were analyzed [APISMAIA, Produtos & Serviços] for botanical origin determination [19,20]. Briefly, 10 g of honey was dissolved in 20 mL of distilled water following centrifugation at 1000× *g* for 10 min. The supernatant was removed and the process was repeated to obtain the honey sediment, which was placed in a slide over a 22 × 22 mm marked square and on a heating plate for drying. Glycerine jelly was applied in a cover slip and placed on the slide and the preparation was heated for 5 min. The relative frequencies, in percentages, of each pollen type were determined by counting grains of pollen in groups of 100, excluding pollen from nectarless plants, following 5 parallel equidistant lines uniformly distributed, until 500 grains were counted. Pollinic types were attributed according to Váldes et al. (1987) [19].

### 2.3. Chemical Characterization of Propolis Extracts and Honey Solutions from Gerês

The chemical characterization of propolis extracts and honey solutions was assessed by three different colorimetric assays: total *ortho*-diphenols, phenolics and flavonoids contents.

#### 2.3.1. Total Ortho-Diphenols Content (TOC)

The content in *ortho*-diphenol compounds was determined using an adaptation of the colorimetric assay proposed by Mateos et al. (2001) [21]. In brief, an identical volume of different concentrations of each of the eleven G.EEs (from 25 to 300 µg/mL) or of each honey sample (H.WSs; from 67,234.0 to 134,467.9 µg/mL) was mixed with 5% (*w*/*v*) Na_2_MoO_4_ in ethanol/water 1:1 (*v*/*v*; following a 4:1 proportion in the reaction mixture), followed by 15 min incubation at room temperature (RT) in the dark. A control and blank were prepared with similar mixtures but with ethanol replacing G.EEs, H.WSs or Na_2_MoO_4_, respectively. The absorbance of the reaction was measured at 370 nm and the results were obtained by interpolation of linear regression using gallic acid as the standard (calibration curve with concentrations ranging from 40 to 180 µg/mL, R^2^ = 1). Total *ortho*-diphenols content was expressed in milligrams of gallic acid equivalent (GAE) per gram of propolis extract or honey (mg GAE/g extract).

#### 2.3.2. Total Phenolics Content (TPC)

The content in phenolic compounds was determined using an adaptation of the Folin–Ciocalteu colorimetric assay [22]. Briefly, different concentrations (from 1 to 300 µg/mL) of each of the eleven G.EEs or (from 1344.7 to 67,234.0 µg/mL) of each honey sample were mixed with 10% (*v*/*v*) Folin–Ciocalteu reagent and 7.5% (*w*/*v*) Na_2_CO_3_ (following a 5:5:4 proportion in the reaction mixture). The control and blank were prepared with similar mixtures but with ethanol replacing G.EEs, H.WSs or reagents, respectively. Absorbance was measured at 760 nm after 1 h of incubation at RT in the dark. The results were obtained by interpolation of linear regression of a gallic acid calibration curve (1 to 50 µg/mL, R^2^ = 1). Total phenolics content was expressed in milligrams of gallic acid equivalent (GAE) per gram of propolis extract or honey (mg GAE/g extract).

#### 2.3.3. Total Flavonoids Content (TFC)

The content in flavonoid compounds was determined using an adaptation of the colorimetric assay previously described by Kumazawa et al. (2004) [22]. In brief, different concentrations (from 200 to 2200 µg/mL) of each of the eleven G.EEs were mixed with 2% (*w*/*v*) AlCl_3_ (following a 1:1 proportion in the reaction mixture). A control and blank were prepared with similar mixtures but with ethanol replacing G.EEs or AlCl_3_, respectively. The absorbance of the reaction was measured at 420 nm after 1 h incubation at RT in the dark. The results were obtained by interpolation of linear regression of a quercetin calibration curve (5 to 200 µg/mL, R^2^ = 0.99). Total flavonoid content was expressed in milligrams of quercetin equivalent (QE) per gram of propolis extract (mg QE/g extract).

### 2.4. In Vitro Evaluation of the Antioxidant Potential of Propolis Extracts and Honey Solutions from Gerês

The DPPH^•^ (2,2-diphenyl-1-picryl-hydrazyl) method was used for in vitro evaluation of the antioxidant potential, as previously described [12]. Succinctly, different concentrations (from 1 to 50 µg/mL) of each G.EE (from 700 to 45,000 µg/mL) or H.WSs were mixed with 0.004% (*w*/*v*) DPPH^•^ (in a proportion of 1:2). The control and blank were prepared with similar mixtures but with ethanol replacing G.EEs, H.WSs or DPPH^•^, respectively. The absorbance of the reaction was measured at 517 nm after 20 min incubation at RT in the dark. The results were expressed in EC_50_ values (µg/mL; concentration that reduces the free radical by 50%) obtained by interpolation from linear regression analysis of the percentage decrease in absorbance with respect to control values.

### 2.5. Antimicrobial Properties of Propolis Extracts and Honey Solutions from Gerês

Minimum inhibitory concentrations (MICs) were determined using the agar dilution method [23] and the following panel of susceptible indicator strains: the Gram-negative bacterium *Escherichia coli* CECT 423, six Gram-positive bacteria (*Bacillus subtilis* 48886, *Bacillus cereus* ATCC 7064, *Bacillus megaterium* 932, (Methicillin-Sensitive) *Staphylococcus aureus* ATCC 6538 (MSSA), Methicillin-Resistant *Staphylococcus aureus* M746665 (MRSA) and *Propionibacterium acnes* H60803), and two yeasts, *Saccharomyces cerevisiae* BY4741 from Euroscarf (http://euroscarf.de/index.php?name=Description (accessed on 9 January 2017)) and *Candida albicans* 53B, obtained from the microbial collection of the Department of Biology of the University of Minho.

Bacteria and yeast were cultured, respectively, in LB (Luria–Bertani—0.5% *w*/*v* yeast extract, 1% *w*/*v* tryptone, 1% *w*/*v* NaCl) and YPD (1% *w*/*v* yeast extract, 2% *w*/*v* peptone, 2% *w*/*v* glucose) broths or solid media (LBA and YPDA—obtained by adding 2% *w*/*v* agar to each of the previous LB and YPD recipes, respectively). Growth was performed at 200 rpm—at 37 °C for bacteria or 30 °C for yeast—and monitored by optical density at 600 nm (OD_600_). To prepare the cells for the experiments, overnight microbial cultures were diluted with the appropriated fresh medium to an OD_600_ = 0.1 and cultured under the same conditions until OD_600_ = 0.4–0.6 (mid-exponential phase).

Drops of 5 µL of exponentially growing microbial cultures were transferred onto LBA and YPDA plates for bacteria and yeast, respectively, supplemented with different concentrations of each of the eleven G.EEs (0, 10, 50, 100, 200, 500, 750, 1000, 1250, 1500, 1750 or 2000 µg/mL) or different concentrations of each of the H.WSs (from 33,617.0 up to 537,871.6 µg/mL, with increments of 33,617.0 µg/mL). Plates containing identical volumes of ethanol instead of the respective extract were used as controls. Plates were incubated at 37 °C for 24 h for bacteria and at 30 °C for 48 h in the case of yeast. MIC values were determined upon observation of the presence/absence of growth.

### 2.6. Statistical Analysis

Chemical profiles by colorimetric assays as well as antioxidant and antimicrobial experiments were performed in triplicate and repeated at least three times independently, with the results being expressed as mean ± standard deviation (SD). One-way ANOVA followed by Tukey’s test for multiple comparisons was used to assess the treatment effect. Differences considered statistically significant (*p* < 0.05) were noted with different letters.

## 3. Results

### 3.1. Botanical Origin of Gerês Honey Samples

The pollen profiles obtained for the Gerês honey samples studied in the present work are presented in Table 1. *Erica* spp. was the most predominant pollen in both samples, representing 44–55% of the total pollen composition, but, while *Castanea sativa* was a secondary pollen for the H17 sample, *Frangula alnus* was the secondary pollen for H18. Nevertheless, H17 and H18 can be classified as heather honey as they present similar botanical origins according to their pollen analysis [24,25].

### 3.2. Total Ortho-Diphenols and Phenolics Contents from Gerês Honey Samples

Total *ortho*-diphenols (TOC) and phenolics (TPC) contents of the honey solutions from Gerês—H17.WS and H18.WS—are presented in Table 2. H18.WS showed the highest content of ortho-diphenol compounds. On the other hand, H17.WS exhibited a higher content of phenolic compounds.

### 3.3. Antioxidant Potential of the Honey Solutions of Gerês 

The antioxidant capacity of the honey solutions from Gerês was evaluated by the DPPH free-radical-scavenging assay and the respective EC_50_ values were calculated. Both H.WSs showed the ability to scavenge the DPPH free radical and, despite both being classified as heather honey, EC_50_ values were significantly different—9662.3 and 5167.5 µg/mL for H17.WS and H18.WS, respectively.

### 3.4. Antimicrobial Properties of the Honey Solutions of Gerês

The antibacterial activity of the honey solutions was evaluated against a panel of susceptibility indicator bacteria (Table 3 and Table 4). The Gram-positive bacteria of the genus *Bacillus* were the most susceptible strains to both extracts. H18.WS was the most effective extract against all the tested strains and the only effective extract against both tested yeasts, with MIC values of 235,318.8 and 302,552.8 µg/mL for *Saccharomyces cerevisiae* and *Candida albicans*, respectively.

### 3.5. Total Ortho-Diphenols, Phenolics and Flavonoids Contents of Gerês Propolis Samples

Table 5 summarizes the total *ortho*-diphenols (TOC), phenolics (TPC) and flavonoids (TFC) contents of different extracts of propolis samples from Gerês. G-r17.EE, G18.EE and G-r18.EE presented the highest contents of *ortho*-diphenol, phenolic and flavonoid compounds. The highest TOC, TPC and TFC were achieved for extracts of propolis sampled only from Roca, while the lowest contents were obtained, in general, from extracts without Roca propolis in their composition. The exception was G18.EE, with the highest TPC value (185.5 ± 7.1 mg GAE/g of propolis). Thus, the addition of Roca as a new source place to collect propolis proves to be an asset, enabling extracts with higher contents of *ortho*-diphenol, phenolic and flavonoid compounds to be obtained.

### 3.6. Antioxidant Potential of the Ethanol Extracts of Gerês Propolis Samples

The antioxidant capacity of the different propolis extracts from Gerês was evaluated by the DPPH free-radical-scavenging assay and the respective EC_50_ values were calculated (Table 6). All propolis extracts showed the ability to scavenge the DPPH free radical, with EC_50_ values ranging from 3.6 to 30.5 µg/mL for G-r17.EE and G-r19.EE, respectively. Propolis samples from 2017, particularly those with propolis from Roca in their composition, exhibited a significantly higher antioxidant potential when compared to the remaining tested samples (EC_50_ = 3.6 µg/mL for G-r17.EE and EC_50_ = 7.1 µg/mL for G-rt17.EE). Interestingly, besides exhibiting the highest antioxidant potential, G-r17.EE was the sample with a significantly higher content of *ortho*-diphenol compounds, suggesting that *ortho*-diphenols could promote antiradical capacity. 

### 3.7. Antimicrobial Properties of the Ethanol Extracts of Gerês Propolis Samples

The antibacterial activity of the eleven propolis extracts was evaluated against a panel of susceptibility indicator bacteria (Table 7). As reported by several authors [18,26], including our research group [12], Gram-positive bacteria, such as the ones of the genus *Bacillus*, were the most susceptible strains to all the extracts. G18.EE was the most effective extract against all the tested strains, followed by the extracts of propolis collected in 2017 (G-r17.EE, G-rt17.EE and G-tf17.EE) and G-tf18.EE and G-rt19.EE, which equally affected the growth of the tested bacteria (Table 7). All the extracts exhibited the same MIC values against the MSSA, MRSA, *Propionibacterium acnes* and *Escherichia coli*, except G-tf17.EE and G18.EE, which were the most active against the Gram-positive *Propionibacterium acnes* (MIC = 200 μg/mL) and MSSA (MIC = 200 μg/mL), respectively. G-tf19.EE was the least active extract, exhibiting higher MIC values for the *Bacillus* strains (MIC = 200 μg/mL). 

The antifungal effect of the eleven extracts was also evaluated (Table 8). All the extracts showed similar activity against both strains, with G-r17.EE and G18.EE standing out from the others as the most active against *Saccharomyces cerevisiae* (MIC = 1500 μg mL^−1^) and G-rt18.EE and G-rt19.EE being the most active against *Candida albicans* (MIC = 1500 μg mL^−1^).

## 4. Discussion

Portuguese beehive products, such as honey and propolis, have been studied by several authors [8,27,28,29,30,31], particularly regarding their phenolic composition and antioxidant and antimicrobial activities. The samples from the present study showed high percentages of *Erica* sp. pollens (Table 1), giving the classification of heather honey to both H17 and H18. Heather honey is characterized by its dark brown color and strong flavor with a slightly salty taste, which is very common in Portugal, being the designation of heather honey most attributed to the studied Portuguese honey samples [32,33,34].

Honey samples from the North of Portugal have been a major object of study, both in northeast and northwest regions [30,33,34,35,36,37,38]. An important aspect of honey is its content in bioactive compounds. The *ortho*-diphenols content of honey is an important parameter to analyze considering its potential influence on propolis bioactivities. Total phenolics contents of Portuguese honey samples have been investigated, showing lower TPC values—between 0.744 and 1.910 mg GAE/g [29,34]—than the results obtained in the present work. Heather honeys from other origins show TPC values between the same range—0.887 (Estonia honey) and 0.110 (Algeria/Babors Kabylia) mg GAE/g [39,40]. Honey phenolic composition not only depends on the main source of its botanical origin but also on the overall interaction of all the plant sources of the honey composition, explaining the difference between the TPC values found for honeys with the same main botanical source [41]. The antioxidant activity of Portuguese honey has been demonstrated by other authors by the ability to scavenge different free radicals, such as the DPPH, peroxyl and nitric oxide, and by the ability to chelate metal ions [35,42]. Between the two honey samples tested in the present work, H18.WS exhibited higher antioxidant potential, suggesting that *ortho*-diphenols could promote radical scavenging capacity as well. Likewise, H18.WS showed greater antimicrobial activity against both bacteria and fungi strains.

Despite both H17 and H18 being classified as heather honey, H18 possesses a considerably higher percentage of *Frangula alnus* pollen in its composition. *F*. *alnus* antioxidant capacity and antimicrobial activity, particularly against *S. aureus*, *E. coli* and *C. albicans*, have been demonstrated [43,44,45,46]. *F*. *alnus* is known to sprout quickly after a fire and its abundance naturally increases with time after a fire [47], suggesting that the H18 chemical composition could have been affected by the increased abundance of this shrub-tree after the occurrence of the wildfires in 2017, providing H18 with greater antioxidant and antimicrobial potential.

Other authors [33,35,37], like what we observed in the present study, found that increasing concentrations of heather honey were able to reduce the growth of yeasts such as *C. albicans* and *S. cerevisiae* and bacteria like *B. subtilis*, *S. aureus* and *E. coli*, with Gram-positive bacteria being more sensitive than Gram-negative bacteria to honey phenolic compounds extracts.

There are only a few studies regarding the TPC and TFC of Portuguese propolis, especially concerning the latter parameter [3,27,31,48,49]. A Portuguese propolis sample from Côa (Beira Alta) in 2010 showed a TFC value (30.2 mg QE/g of propolis) similar to the one obtained in the present study for G-r17.EE [48]. Different propolis samples from Pereiro [31] also showed similar values for both TFC and TPC when compared to the samples tested in our work (Table 5). Propolis from Bornes, Côa and Fundão presented significant differences between their TPC values (329, 160.4 and 151 mg GAE/g of propolis, respectively), with Côa [48] and Fundão [3] propolis exhibiting TPC values closer to the ones found for Gerês samples, particularly the ones with Roca propolis in their composition. TPC values ranging from 143.0 ± 1.9 (for G11.EE) to 212.2 ± 9.2 (G12.EE) mg GAE/g of propolis and TFC values ranging from 31.0 ± 1.4 (for G14.EE) to 51.7 ± 0.9 (for G15.EE) mg QE/g of propolis were obtained for Gerês propolis samples from previous years (G11.EE-G16.EE) [49]. Still, the TPC and TFC values obtained in the present work were in the range of those usually observed not only in Gerês propolis samples but also in propolis samples worldwide [50]. All these results demonstrate that TPC and TFC parameters are typically little affected by geographic origin.

The antioxidant capacity of different propolis samples from Portugal was demonstrated using the DPPH free-radical-scavenging assay [8,12,27,51,52]. Two propolis samples, Fundão (center of Portugal) [3] and Algarve (south of Portugal) [8], presented a significantly lower capability to reduce DPPH radicals (EC_50_ of 52 and 40 µg/mL, respectively) when compared to extracts tested in the present work (Table 2). Even though, and similarly to our results, different Portuguese propolis samples—from the north (Bornes), center (Leiria) and south (Algarve)—exhibited higher DPPH radical scavenging activity, with EC_50_ values ranging from 6 to 31 µg/mL [8,27,51]. The EC_50_ values obtained in the present work are in line with those obtained for Gerês propolis extracts from previous years (G11.EE-G16.EE) made with a mixture of all places of the apiary (Bugalho, Felgueiras and Toutelo), with values ranging from 5.5 to 25.2 µg/mL for G16.EE and G13.EE, respectively [12,49]. Additionally, and like what was found for the TOC, TPC and TFC contents, the region does not seem to influence the antioxidant capacity of Portuguese propolis, as different samples from the same region can exhibit distinct DPPH free-radical-scavenging capacity. Other samples from different origins were found to exhibit significantly lower DPPH radical scavenging activity as the EC_50_ values were found to be at least ten times higher than the ones found in the present study, ranging from 630 for Uruguay propolis to 325 µg/mL for Turkish propolis [50].

The antimicrobial activity of propolis has been studied for samples from different Portuguese regions, showing considerable activity against several bacterial and fungal strains [12,28,30,52,53,54]. Propolis samples from the Trás-os-Montes region displayed similar antimicrobial activity against MRSA despite their different pollen types, suggesting that this activity is independent of propolis botanical origin [53]. Despite the lower activity of some extracts (G-r18.EE, G-rt18.EE, G-r19.EE and G-tf19.EE) against the bacteria of the genus *Bacillus*, in general, extracts from propolis collected in 2017 (G-r17.EE, G-rt17.EE and G-tf17.EE) and G-tf18.EE and G-rt19.EE exhibited similar activity to the G.EEs of previous years, which were prepared from a mixture of Bugalho, Felgueiras and Toutelo samples [12,52]. However, only G18.EE exhibited exactly the same activity spectrum as extracts of propolis from Gerês, pointing to the importance of Bugalho, in combination with Toutelo and Felgueiras, to reach the greatest bioactive potential. Still, this work supports the importance of mixing different propolis samples, even from the same apiary, to normalize propolis chemical and biological profiles [49], which is of utmost importance considering propolis scarcity and the lack of standardization. With the exception of the above-mentioned G.EEs, propolis extracts displayed similar antifungal activity as propolis from the same apiary from previous years (G11.EE–G16.EE), presenting MICs above 2000 µg/mL [12,52]. Similarly, a propolis sample from Ireland showed low MIC values against *B*. *subtillis* (80 µg/mL), while samples from other origins, such as Germany and the Czech Republic, exhibited a much higher MIC value (300 µg/mL) [55,56]. The same was observed against *S*. *aureus*, with propolis samples exhibiting lower MIC values (8 and 10 µg/mL for Turkey and Taiwan propolis, respectively) and others displaying more than 100 times this value (1200 and 1445 µg/mL for Australia and Chile propolis, respectively) [56].

## 5. Conclusions

Propolis has been the subject of intensive studies owing to its long and widely recognized remarkable properties. The first study of Portuguese propolis dates only from 2008, but its great potential is being demonstrated over and over. However, Portuguese propolis is still undervalued and often discarded; it is far from receiving its due attention from beekeepers and the international scientific community, despite its remarkable properties. Once again, propolis from Gerês showed antioxidant and antimicrobial potential, regardless of apiary sources and harvesting years. Some differences were found in the bioactivities of the tested extracts composed of different propolis sources when compared to the extracts of propolis samples harvested from previous years (G11.EE–G16.EE) made from a mixture of propolis originating from Bugalho, Felgueiras and Toutelo. The differences regarding both antioxidant and antimicrobial activity were more evident after the occurrence of the wildfire, which resulted in the relocation of some of the beehives, with DPPH radical scavenging activity being around ten times lower after the natural disaster. The same decrease in antimicrobial activity was observed, particularly against the Gram-positive spore-forming bacteria of the genus *Bacillus*. These results show the significant impact that the relocation of beehives, as a result of the environmental disaster, can have on beehive product composition and, consequently, bioactivities. In addition, this work highlights the importance of the constancy of a combination of aspects, such as the preservation of the plant species around hives, to provide bee products with more constant and reliable chemical and biological profiles over time, making it possible to establish a chemical and biological fingerprint. The results also show that mixing different propolis samples can contribute to a dilution of the differences between samples, helping to overcome the low production of propolis. Still, this thorough characterization of propolis samples from a single apiary, which operates in an organic mode, opens doors to a more global understanding of the chemical and biological properties of hive products from a single producer. Thus, this work is a contribution to the future of production propolis with more constant properties.

## Figures and Tables

**Table 1:** Botanical origin (%) of Gerês honey samples H17 and H18 collected in 2017 and 2018, respectively.

Species	Pollen Composition (%)	
	H17	H18
*Erica* spp.	55	44
*Frangula alnus*	0	18
*Castanea sativa*	20	13
*Echium plantagineum*	5	8
*Campanula* spp.	0	5
*Rubus* spp.	7	2
Others	13	10

**Table 2:** Total *ortho*-diphenols (TOC) and phenolics (TPC) contents of H17.WS and H18.WS. Results are presented as mean ± standard deviation (SD) of mg of gallic acid equivalent per g of extract (mg GAE/g extract). Statistical analysis was performed by one-way ANOVA followed by Tukey test for multiple comparisons. For each individual parameter (TOC and TPC), different letters (a or b) mean statistically significant differences between mean values.

Honey Solutions	Total *Ortho*-Diphenols Content	Total Phenolics Content
	mg GAE/g	mg GAE/g
H17.WS	0.455 ± 0.005 ^a^	3.079 ± 0.145 ^a^
H18.WS	0.535 ± 0.011 ^b^	2.438 ± 0.035 ^b^

**Table 3:** G.EE MIC values (µg/mL) obtained from the panel of susceptibility indicator bacteria. Mid-exponential-phase bacterial cultures were transferred to plates supplemented with increasing concentrations of each of the honey solutions—H17.WS and H18.WS. Plates were observed for the presence/absence of growth after 24 h incubation at 37 °C, and the lowest concentrations for which no growth was detected were registered as the MIC.

Honey Solutions	Antibacterial Activity						
	*B. subtilis*	*B. cereus*	*B. megaterium*	MSSA	MRSA	*P. acnes*	*E. coli*
	MIC (µg/mL)						
H17.WS	268,935.8	302,552.8	268,935.8	537,871.6	369,786.7	302,552.8	369,786.7
H18.WS	168,084.9	134,467.9	201,801.9	235,318.8	235,318.8	235,318.8	235,318.8

MSSA—Methicillin-Sensitive *Staphylococcus aureus*; MRSA—Methicillin-Resistant *Staphylococcus aureus*.

**Table 4:** G.EE MIC values (µg/mL) obtained from the two susceptibility indicator yeasts. Mid-exponential-phase yeast cultures were transferred to plates supplemented with increasing concentrations of each of the honey solutions—H17.WS and H18.WS. Plates were observed for the presence/absence of growth after 48 h incubation at 30 °C, and the lowest concentrations for which no growth was detected were registered as the MIC.

Honey Solutions	Antifungal Activity	
	*Saccharomyces cerevisiae*	*Candida albicans*
	MIC (µg/mL)	
H17.WS	>537,871.6	>537,871.6
H18.WS	235,318.8	302,552.8

**Table 5:** Total *ortho*-diphenols (TOC), phenolics (TPC) and flavonoids (TFC) contents of G-r17.EE, G-rt17.EE, G-tf17.EE, G-r18.EE, G-rt18.EE, G-tf18.EE, G18.EE, G-r19.EE, G-rt19.EE, G-tf19.EE and G19.EE. Results are presented as mean ± standard deviation (SD) of mg of gallic acid equivalent per g of extract (mg GAE/g extract) for TOC and TPC and of mg of quercetin equivalent per g of extract (mg QE/g extract) for TFC. Statistical analysis was performed by one-way ANOVA followed by Tukey test for multiple comparisons. For each individual parameter (TOC, TPC and TFC), the same letters (a, b, c, d, e or f) mean no statistically significant differences between mean values.

Propolis Extracts	Total *Ortho*-Diphenol Content	Total Phenolic Content	Total Flavonoid Content
	mg GAE/g	mg GAE/g	mg QE/g
G-r17.EE	467.2 ± 13.1 ^a^	177.9 ± 6.7 ^a^	36.8 ± 2.3 ^a^
G-rt17.EE	271.0 ± 3.9 ^b^	130.9 ± 9.0 ^b^	22.5 ± 1.8 ^b^
G-tf17.EE	243.5 ± 6.8 ^b,d^	125.1 ± 5.5 ^b,d^	30.5 ± 1.1 ^c^
G-r18.EE	373.8 ± 19.3 ^c^	148.4 ± 4.1 ^c^	44.8 ± 2.8 ^d^
G-rt18.EE	295.7 ± 51.8 ^b^	82.9 ± 5.3 ^e^	29.6 ± 1.6 ^c,f^
G-tf18.EE	205.6 ± 17.2 ^d,e^	73.7 ± 6.9 ^e^	25.7 ± 1.9 ^b,f^
G18.EE (G-tfb18.EE)	270.0 ± 15.2 ^b^	185.5 ± 7.1 ^a^	35.8 ± 0.5 ^a,e^
G-r19.EE	285.9 ± 14.3 ^b^	136.2 ± 5.3 ^b,c^	30.7 ± 0.8 ^c^
G-rt19.EE	299.3 ± 9.3 ^b^	135.6 ± 9.4 ^b,c^	29.0 ± 0.8 ^c,f^
G-tf19.EE	247.7 ± 15.9 ^b,e^	110.3 ± 5.9 ^d^	25.6 ± 0.9 ^b,f^
G19.EE (G-rtf19.EE)	272.3 ± 4.7 ^b^	135.2 ± 8.2 ^b,c^	31.9 ± 1.1 ^c,e^

EE—ethanol extract; r—Roca; t—Toutelo; f—Felgueiras; b—Bugalho.

**Table 6:** Antioxidant potential of G-r17.EE, G-rt17.EE, G-tf17.EE, G-r18.EE, G-rt18.EE, G-tf18.EE, G18.EE, G-r19.EE, G-rt19.EE, G-tf19.EE and G19.EE—measured by the in vitro DPPH free-radical-scavenging assay and expressed as a mean of EC_50_ values (μg/mL) and respective standard deviation (SD). Statistical analysis was performed by one-way ANOVA followed by Tukey test for multiple comparisons. The same letters (a, b, c, d, e, f, g or h) mean no statistically significant differences between mean values.

Propolis Extracts	DPPH^•^ Scavenging Activity
	EC_50_ (µg/mL)
G-r17.EE	3.6 ± 0.3 ^a^
G-rt17.EE	7.1 ± 0.8 ^b^
G-tf17.EE	20.9 ± 0.1 ^c^
G-r18.EE	20.1 ± 0.1 ^c,e^
G-rt18.EE	19.0 ± 0.5 ^e,f^
G-tf18.EE	18.3 ± 0.7 ^f^
G18.EE (G-tfb18.EE)	12.4 ± 0.4 ^d^
G-r19.EE	30.5 ± 0.3 ^g^
G-rt19.EE	19.5 ± 1.3 ^c,f^
G-tf19.EE	21.2 ± 0.5 ^c^
G19.EE (G-rtf19.EE)	24.3 ± 0.1 ^h^

EE—ethanol extract; r—Roca; t—Toutelo; f—Felgueiras; b—Bugalho.

**Table 7:** G.EE MIC values (μg/mL) obtained from the panel of susceptibility indicator bacteria. Mid-exponential-phase bacterial cultures were transferred to plates supplemented with increasing concentrations of each of the eleven G.EEs—G-r17.EE, G-rt17.EE, G-tf17.EE, G-r18.EE, G-rt18.EE, G-tf18.EE, G18.EE, G-r19.EE, G-rt19.EE, G-tf19.EE and G19.EE. Plates were observed for the presence/absence of growth after 24 h incubation at 37 °C, and the lowest concentrations for which no growth was detected were registered as the MIC.

Propolis Extracts	Antibacterial Activity						
	*B. subtilis*	*B. cereus*	*B. megaterium*	MSSA	MRSA	*P. acnes*	*E. coli*
	MIC (µg/mL)						
G-r17.EE	50	50	50	500	>2000	500	>2000
G-rt17.EE	50	50	50	500	>2000	500	>2000
G-tf17.EE	50	50	50	500	>2000	200	>2000
G-r18.EE	100	100	200	500	>2000	500	>2000
G-rt18.EE	100	100	100	500	>2000	500	>2000
G-tf18.EE	50	50	50	500	>2000	500	>2000
G18.EE (G-tfb18.EE)	50	50	50	200	>2000	500	>2000
G-r19.EE	100	100	100	500	>2000	500	>2000
G-rt19.EE	50	50	50	500	>2000	500	>2000
G-tf19.EE	200	200	200	500	>2000	500	>2000
G19.EE (G-rtf19.EE)	100	100	100	500	>2000	500	>2000

MSSA—Methicillin-Sensitive *Staphylococcus aureus*; MRSA—Methicillin-Resistant *Staphylococcus aureus*; EE—ethanol extract; r—Roca; t—Toutelo; f—Felgueiras; b—Bugalho.

**Table 8:** G.EE MIC values (μg/mL) obtained from the two susceptibility indicator yeasts. Mid-exponential-phase yeast cultures were transferred to plates supplemented with increasing concentrations of each of the eleven G.EEs—G-r17.EE, G-rt17.EE, G-tf17.EE, G-r18.EE, G-rt18.EE, G-tf18.EE, G18.EE, G-r19.EE, G-rt19.EE and G19.EE. Plates were observed for the presence/absence of growth after 48 h incubation at 30 °C, and the lowest concentrations for which no growth was detected were registered as the MIC.

Propolis Extracts	Antifungal Activity	
	*Saccharomyces cerevisiae*	*Candida albicans*
	MIC (µg/mL)	
G-r17.EE	1500	>2000
G-rt17.EE	>2000	>2000
G-tf17.EE	>2000	>2000
G-r18.EE	2000	2000
G-rt18.EE	>2000	1500
G-tf18.EE	>2000	2000
G18.EE (G-tfb18.EE)	1500	2000
G-r19.EE	>2000	2000
G-rt19.EE	>2000	1500
G-tf19.EE	>2000	2000
G19.EE (G-rtf19.EE)	>2000	2000

EE—ethanol extract; r—Roca; t—Toutelo; f—Felgueiras; b—Bugalho.

## Data Availability

Data are contained within the article.
